# High-throughput metabolomics enables metabolite biomarkers and metabolic mechanism discovery of fish in response to alkalinity stress†

**DOI:** 10.1039/c8ra01317a

**Published:** 2018-04-19

**Authors:** Yan-chun Sun, Song Wu, Ning-ning Du, Yi Song, Wei Xu

**Affiliations:** Heilongjiang River Fisheries Research Institute of Chinese Academy of Fishery Sciences/Laboratory of Quality & Safety Risk Assessment for Aquatic Products (Harbin), Ministry of Agriculture Harbin 150070 China Sunyc2004@163.com +86-0451-84604803 +86-0451-84861316; Chinese Academy of Fishery Sciences Beijing 100141 P. R. China

## Abstract

High throughput mass spectrometry (MS)-based metabolomics is a popular platform for small molecule metabolites analyses that are widely used for detecting biomarkers in the research field of environmental assessment. Crucian carp (*Carassius carassius*, CC) is an economically and ecologically important fish in Asia. It can adapt to extremely high alkalinity, providing us with valuable material to understand the adaptation mechanism for extreme environmental stress. However, the information on the metabolite biomarkers and metabolic mechanisms of CC exposed to alkaline stress is not entirely clear. We applied high-throughput UPLC-Q-TOF/MS combined with chemometrics to identify changes in the metabolome of CC exposed to different concentrations of alkalinity for long term effects. Metabolic differences among alkalinity-treated groups were identified by multivariate statistical analysis. Further, 7 differential metabolites were found after exposure to alkaline conditions. In total, 23 metabolic pathways of these differential metabolites were significantly affected. Alkalinity exposure resulted in widespread change in metabolic profiles in the plasma with disruptions in the phenylalanine metabolism, glycine, serine and threonine metabolism, pyruvate metabolism, tyrosine metabolism, *etc.* The integrated pathway analysis of the associated metabolites showed that tRNA charging, l-cysteine degradation II, superpathway of methionine degradation, l-serine degradation, tyrosine biosynthesis IV, *etc.* appear to be the most significantly represented functional categories. Overall, this study demonstrated that metabolic changes in CC played a role in adaptation to the highly alkaline environmental stress.

## Abstract 2

High throughput mass spectrometry (MS)-based metabolomics is a popular platform for small molecule metabolites analyses that are widely used for detecting biomarkers in the research field of environmental assessment.

## Graphical abstract

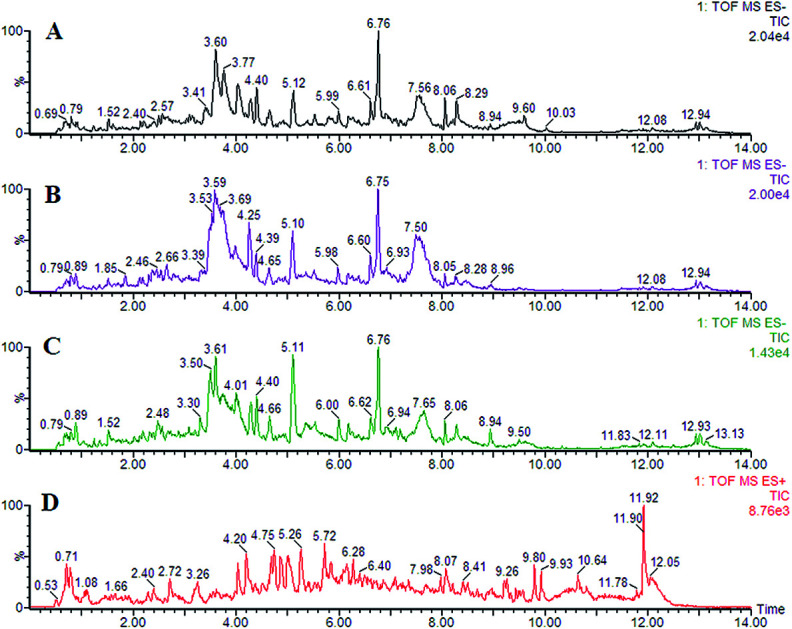

## Introduction

Crucian carp (also abbreviated CC) belongs to the genus of *Carassius* within the family of Cyprinidae.^1^ It has been part of the main freshwater species for aquaculture and has been widely used as a raw material for food production.^2,3^ Scientists are paying more attention to CC as it is gaining increasing significance economically and ecologically.^4^ CC has been recently developed as a potential aquaculture species in the widely distributed alkaline water. Although CC inhabits fresh water in streams, rivers, and lakes, it also has great tolerance to high alkalinity.^5^ In spite of its economic and ecological importance, the metabolic mechanism of its extreme tolerance to alkaline conditions is still a puzzle. A better understanding of the metabolic basis of the tolerance adaptation and resistance to high alkaline environment is desired.

Metabolomics, which involves the unbiased identification of small molecules in biological fluids, can be used as an approach to understand the biochemical state of an organism and aiding in the biomarker discovery.^6^ Recently, metabolomics has identified a wide diversity of small molecules in the plasma, which can provide information for identifying potential biomarkers.^7–10^ The high-throughput MS metabolomics platform was performed and analyzed, providing the metabolic basis for further investigation of the mechanism of the high alkaline tolerance.^11,12^ An integrated analytical approach based on high resolution ultra performance liquid chromatography-mass spectrometry (UPLC-MS) combined with pattern recognition approach can simultaneously investigate any associated disruption of the metabolism.^13^ Recently, we have demonstrated that the detection of differentially expressed metabolites in samples can be enhanced by using high-throughput UPLC-Q-TOF/MS combined with chemometrics.^14–17^

To better understand the physiological changes and the mechanism of elevated alkaline tolerance and adaptation, comparative analysis between the fish living in alkaline water and fresh water is an efficient method. The purpose of this study was to identify differentially expressed metabolites that played a role in adaptation to the complicated and highly alkaline environment and analyzed using UPLC-MS metabolomics approach, followed by chemometric analysis to gain insights into the environmental adaptation mechanism. In the current study, the differentially expressed metabolites were identified and functional annotation and pathway analyses were performed, which provides a valuable resource for unveiling the metabolic mechanism of alkaline tolerance.

## Experimental

### Reagents and materials

Acetonitrile (ACN) and methanol were purchased from Honeywell (Muskegon, MI, USA). Distilled water (18.2 MΩ) was purified using a Milli-Q system (Millipore, Billerica, USA). Leucine enkephalin was acquired from Sigma-Aldrich (St. Louis, MO, USA). Formic acid (HPLC grade) was purchased from J&K Chemical Ltd (Beijing, China). All other chemicals were acquired from Sigma-Aldrich (St Louis, MO, USA) unless otherwise specified.

### Ethics statements

In this study, all animal procedures were performed in accordance with the Guidelines for Care and Use of Laboratory Animals of Heilongjiang River Fisheries Research Institute of Chinese Academy of Fishery Sciences and approved by the Animal Ethics Committee of Heilongjiang River Fisheries Research Institute of Chinese Academy of Fishery Sciences.

### Animals

CC were obtained from a local supplier and acclimatized to lab conditions in 66 L-flow-through tanks (10–16 °C, clear water). During holding, fish were held on a 12 : 12 light : dark photoperiod and fed commercial fish food (TetraMin®, Melle, Germany) every day. The fish were transferred to experimental tanks one week prior to the start of the experiment.

### Alkalinity exposures and sampling

CC (age 2, length of 15.5 ± 1.6 cm and weight 50.2 ± 6.2 g) were distributed among the tanks such that there were 8 fish (four males, four females) per tank during the exposure. The flow rate was 10 L min^−1^ and the tanks were continually aerated with an average temperature of 20 °C ± 1 and a light/dark cycle of 12 h : 12 h. Fish were exposed to either a final treated carbonate alkalinity (CA) or clean water (control) for 60 days. In this experiment, three separate tanks containing carbonate alkalinity (CA) of 20 mmol L^−1^ (CA20), 40 mmol L^−1^ (CA40) and 60 mmol L^−1^ (CA60) were set. Wastewater effluent samples were collected every week of the exposure period and the fish were fed once a day with commercial fish food (TetraMin®, Melle, Germany). All fish appeared healthy during the exposure. Each fish was caught individually in a net and removed slowly in an attempt to minimize capture-induced stress in the caught fish and other fish in the aquarium. Blood was collected from the caudal vein using heparinized tuberculin syringes and placed in centrifuge tubes. Blood samples were centrifuged (10 min, 6000 rpm) and the plasma supernatant was collected. All the plasma samples were stored at −80 °C until analysis.

### UPLC-QTOF/MS analysis

Plasma samples were analyzed by the UPLC-QTOF/MS analyzer equipped with an electrospray ionization (ESI) source. UPLC-QTOF/MS analyses were performed on a UPLC™ BEH C18 column (2.1 × 100 mm, 1.7 μm, Waters, Milford, USA). The column temperature was maintained at 40 °C. The flow rate was 0.4 mL min^−1^ and the sample injection volume was 2 μL for each run. Mobile phases consisted of acetonitrile/water (95 : 5 v/v) containing 0.1% formic acid (solvent A) and water containing 0.1% formic acid (solvent B). Gradient elution was performed with the following solvent system: (A) 0.1% formic acid–water and (B) acetonitrile. The gradient elution was performed as follows: 1–10% A at 0 to 2.0 min, 10–25% A at 2.0 to 6.0 min, 25–55% A at 6.0 to 10.0 min, 55–95% A at 10.0 to 12.0 min, and 95% A at 12.0 to 14.0 min.

Mass spectrometry data acquisition was performed using the quadruple time-of-flight mass spectrometer (Waters, Milford, USA). Each sample was run separately in positive and negative electrospray modes. The TOF mass range was set at *m*/*z* 50–1000 Da in the full scan mode. TOF-MS system was operated with the following settings: source temperature, 110 °C; capillary temperature, 250 °C; desolvation gas temperature, 400 °C; desolvation gas, 500 L h^−1^; cone gas, 50 L h^−1^; cone voltage, 20 V; capillary voltage, 2.5 kV; extraction cone voltage, 5.5 V.

### Data processing and chemometrics analysis

Mass spectra were collected in full scan mode (50–1000 *m*/*z*) and spectral peaks were deconvoluted and aligned using Masslynx 4.1 (Waters, Manchester, UK). The preprocessing results generated a data matrix that consisted of the retention time, mass-to-charge ratio values, and peak intensity. After peak-picking and integration, retention time correction, peak alignment and deconvolution, a two-dimensional data matrix including *R*_t_ variables of the observation samples was generated. These data were fed to the EZinfo 2.0 software for chemometrics analysis, which included unsupervised principal component analysis (PCA) and supervised recognition pattern orthogonal projection to latent structures analysis (OPLS-DA). With an aim to discover the potential variables contributing to the differentiation, we generated an “Important variables on the projection (VIP)” plot for the OPLS-DA model; the VIP plot is used to define metabolites significantly contributing to the separation of groups. MassFragment™ manager (Waters corp., Milford, USA) was used to facilitate the MS/MS fragment ion analysis process. The putative identities of metabolites were determined from screening the *m*/*z* of molecular ions using the online databases of the Human Metabolome Database (http://www.hmdb.ca/), METLIN (http://metlin.scripps.edu/), and LipidMaps (http://www.lipidmaps.org/). Compound annotation was done by comparing the MS/MS spectra and retention time of commercially available standard compounds.

### Metabolic pathway analyses

Metabolic pathway analysis derived the consequences from the pathway enrichment and the topology analysis. The biological function and pathway analysis for the identified metabolites were sought *via* enrichment analysis using online MetaboAnalyst 2.0 (http://www.metaboanalyst.ca). In the context of pathway analysis, the statistical *p* values were further corrected for multiple testing.

### Molecular network analysis

Differentially expressed metabolites were further analyzed using the Ingenuity Pathway Analysis software (IPA; https://analysis.ingenuity.com) in order to identify the affected biochemical pathways. The molecular pathway was identified using the IPA according to specific Knowledge Database. The IPA software can identify global canonical pathways, dynamical biological networks and the functions from a given list of metabolites. The metabolites (HMDB numbers) were imported into IPA and then, “core analysis” was performed, including both the direct and the indirect relationship between our dataset and the reference annotations, in order to interpret data in the context of biological pathways, molecular functions and the networks.

### Statistical analysis

Chemometric analysis was utilized to understand the global metabolic changes with treatment (control or exposed) as factors, and the corresponding VIP values were calculated in the OPLS-DA model. Student's *t*-test analysis was performed using SPSS (Statistical Package for the Social Sciences) 19.0 software (SPSS Inc., U.S.A.), with *p* < 0.05 considered as significant.

## Results

### Typical total ions chromatograms

**Fig. 1:**
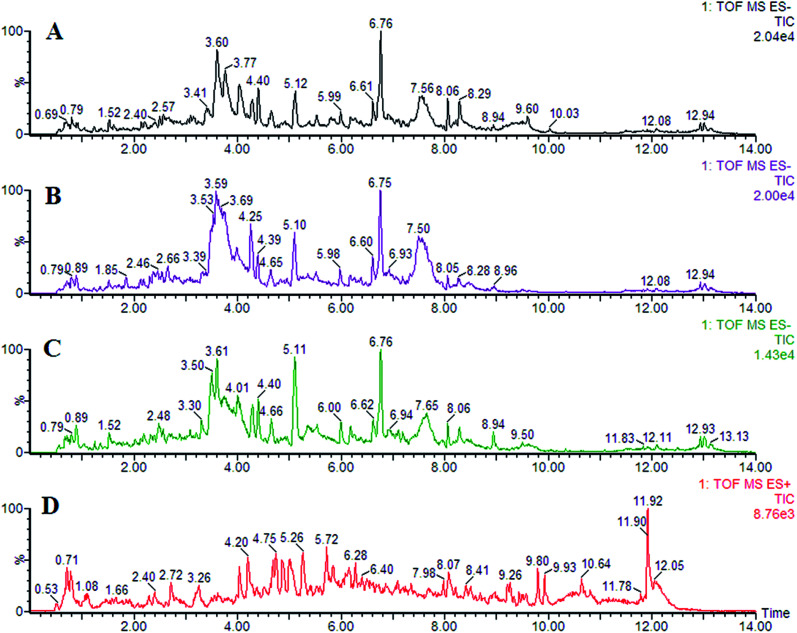
Representative chromatogram of plasma samples obtained as a result of employing the UPLC/MS tool.

In this study, representative chromatograms of plasma samples were analyzed by UPLC-MS. The typical total ion current chromatograms of plasma samples for control or exposed groups in ESI^+^ and ESI^−^ mode are shown in Fig. 1, indicating the stability of the UPLC-MS performance and the reliability of the metabolomics data.

### Metabolic profiles analyses

**Fig. 2:**
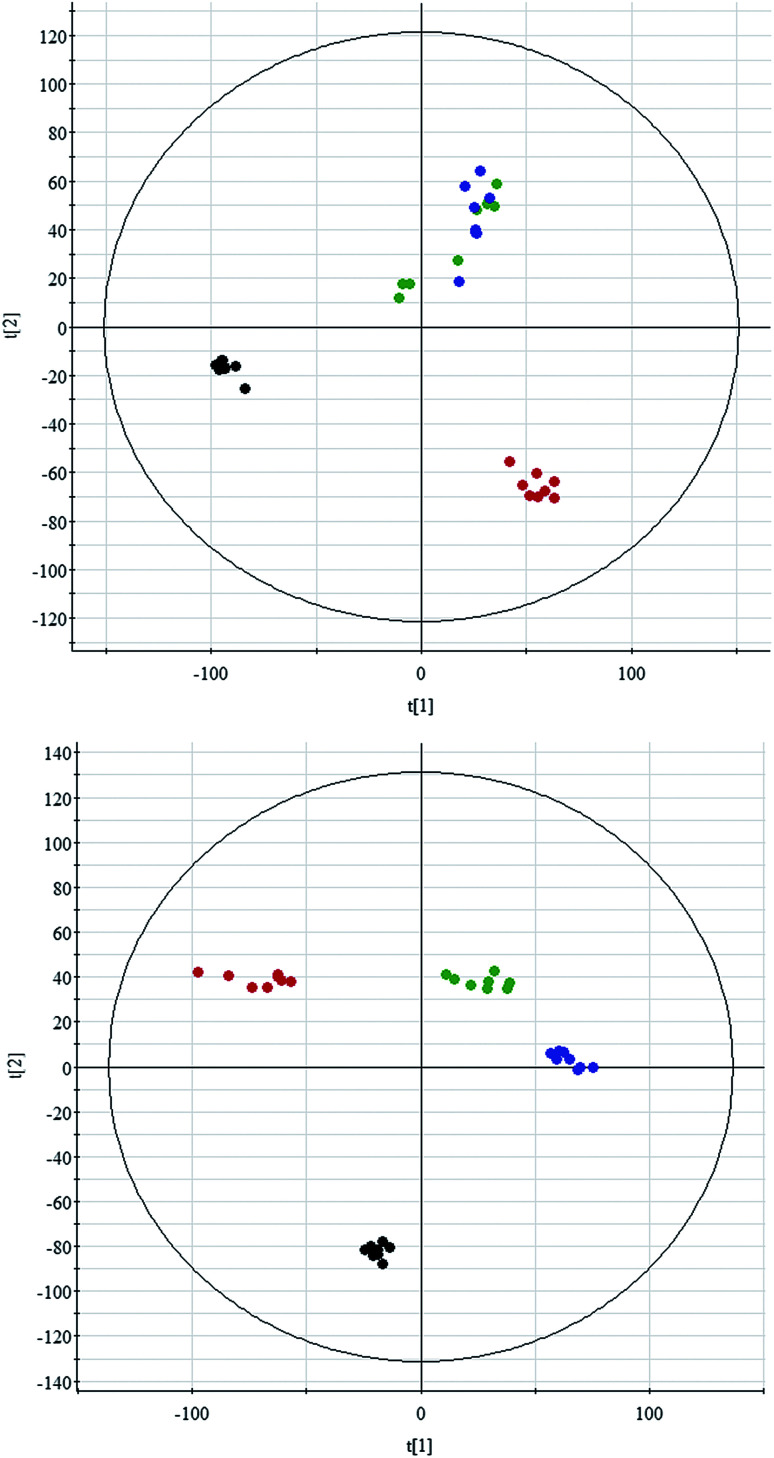
Principal component analysis scores plot of the CC metabolic profiles in positive electrospray mode (up) and negative electrospray modes (down). The black, green, blue and red spots indicate control and carbonate alkalinity of 20 mmol L^−1^, 40 mmol L^−1^, and 60 mmol L^−1^, respectively.

**Fig. 3:**
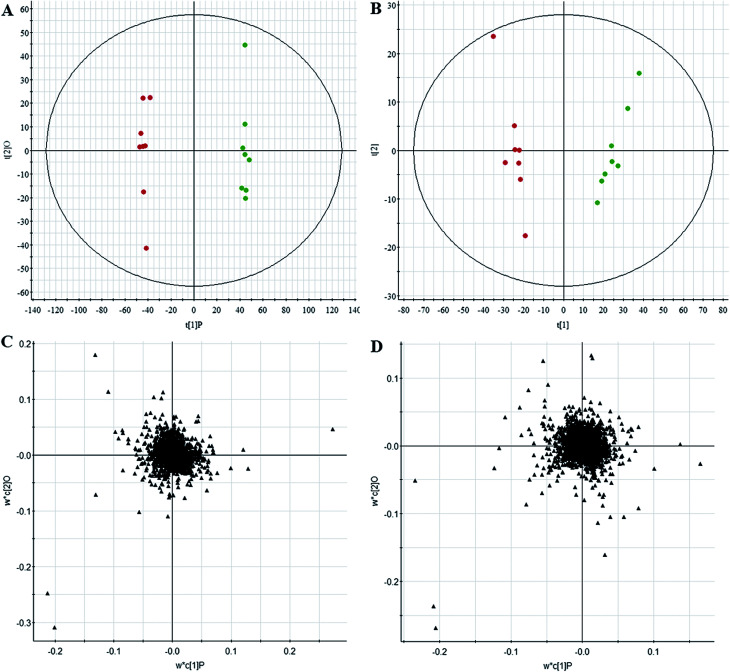
Chemometrics analysis. OPLS-DA score plot of the UPLC/MS data from the CC metabolic profiles in positive electrospray mode (A) and negative electrospray modes (B). Loading plot of the UPLC/MS data in positive electrospray mode (C) and negative electrospray modes (D).

All data on retention time, exact mass, and peak intensity were recorded for multiple statistical analyses, including PCA and OPLS-DA. These analytical methods were chosen because of their ability to deal with multivariate, noisy, collinear, and possibly incomplete data. UPLC-MS results were displayed as “score plots” by PCA, which represent the distribution of the samples in multivariate space. PCA score plots were obtained from the UPLC-MS data for the three exposed groups, and showed that the three groups exhibited different tendencies, indicating their diverse metabolic profiles (Fig. 2). PCA of the data sets revealed clear separation between samples from the control and CA60-exposed groups in the first component of score plots (+ESI mode) and the second component of the scores plots (−ESI mode). After filtering the noise of the OPLS-DA model, the CA60-exposed and control groups were still significantly separated by the primary component axis (Fig. 3A and B). A loading plot (Fig. 3C and D) was constructed based on the OPLS-DA, which showed the contribution of the variables to the differences between the two groups.

### Discovery and identification of differentially expressed metabolites

**Fig. 4:**
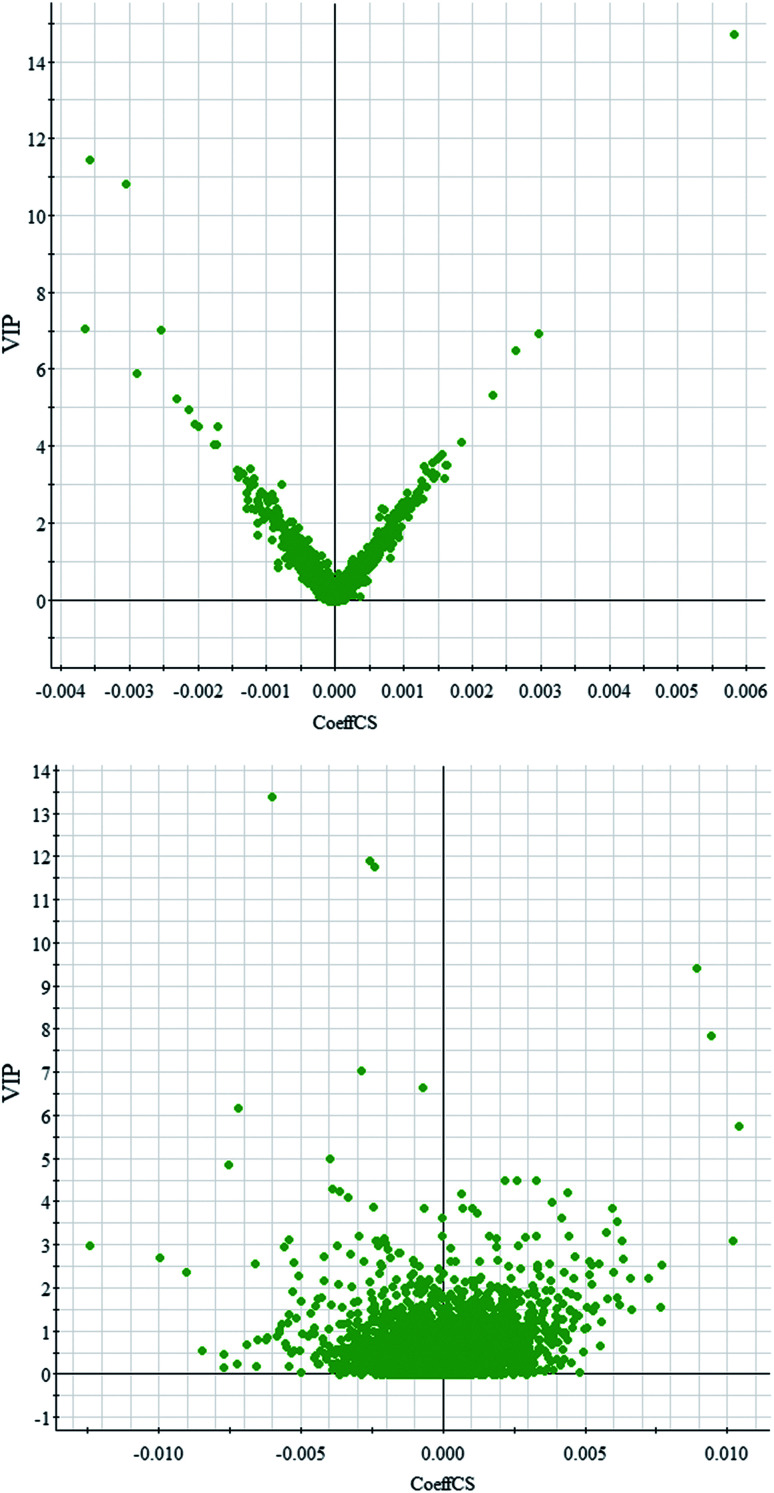
OPLS-DA VIP-plot for screening of differentially expressed metabolites in positive electrospray mode (up) and negative electrospray modes (down).

Comparative analysis among the CA60 and control fish was performed to investigate the different metabolomes. We employed an additional multivariate statistical approach termed VIP-plot to select metabolites that contributed to this group behavior observed by OPLS-DA. Higher values of VIP indicate metabolites that are more important to the classification. Metabolites with VIP scores (Fig. 4) greater than 9 were considered significant. Filtered by the VIP cut-off and *p* value, we obtained 7 differential metabolites. Elemental composition was calculated using the MassFragment™ manager in Masslynx 4.1(Waters corp., Milford, USA). Finally, the obtained elemental composition was confirmed by comparison with that of a standard sample, which was tentatively assigned, based on structural analogs or by matching their accurate mass formula with online databases including METLIN, HMDB and Lipid Map. Eventually, 7 metabolites were tentatively identified as potential biomarkers, namely, l-cysteine, l-tyrosine, phenylpyruvic acid, acetoacetic acid, pyruvic acid, l-phenylalanine, and l-serine and are listed in Table S1.†

### Metabolic pathways and functional analysis

**Fig. 5:**
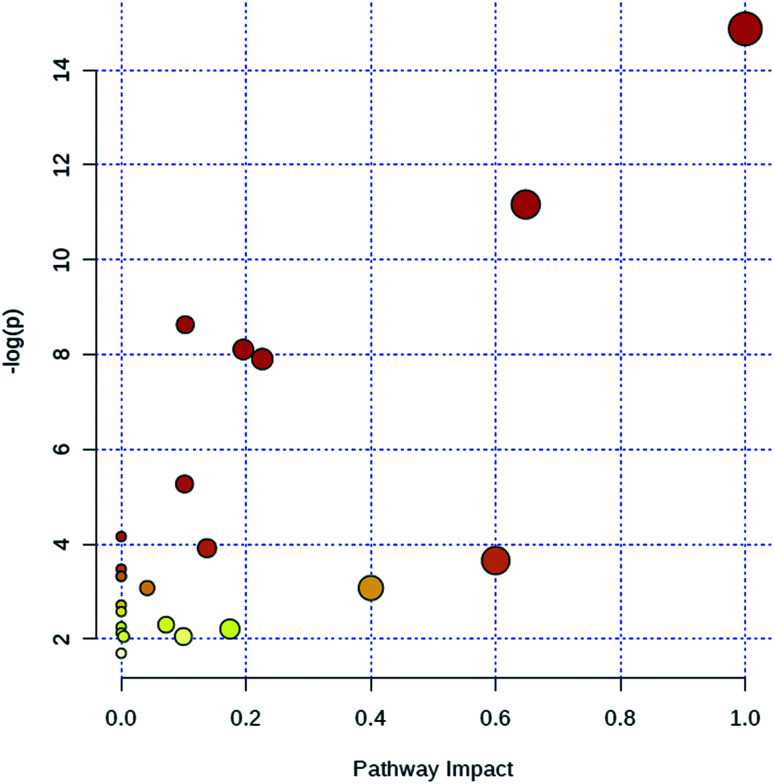
Summary of metabolic pathways of significantly changed metabolites with MetPA. Circles represent metabolism (ESI Table S2†). All matched pathways are plotted depending on *p*-value from pathway enrichment analysis and pathway impact score from pathway topology analysis. Colour gradient and circle size indicate the significance of the pathway ranked by *p*-value (yellow: higher *p*-values and red: lower *p*-values) and pathway impact score (the larger the circle the higher the influence score), respectively.

Through MetaboAnalyst 2.0 analysis, the significant impact pathways were determined with the impact value close to 0.1. MetPA was used to interpret and visualize the metabolome expression profiling data. It allowed us to analyze key pathways of differentially expressed metabolites related to alkalinity stress and identify enriched pathways from the differential expression metabolite data. Pathway analysis was performed on 7 significantly altered plasma metabolites between the control and CA60 exposed CC. The results from the integration of enrichment and pathway topology analyses were utilized to map the identified metabolites into specific metabolic pathways. On this basis, 23 pathways were thought to be potentially affected during the alkalinity stress (Fig. 5, ESI Table S2†). The phenylalanine metabolism was the most significantly affected pathway based on *p*-values and pathway impact scores. Other impacted pathways included glycine, serine and threonine metabolism, pyruvate metabolism, tyrosine metabolism, cysteine and methionine metabolism, aminoacyl-tRNA biosynthesis, pyruvate metabolism, and butanoate metabolism. Most of the key intermediate metabolites involved in these metabolisms were significantly altered during the alkalinity stress. Collectively, pathways identified herein may cause integral disturbance on CC under alkaline stress.

### Integrated pathway analyses

**Fig. 6:**
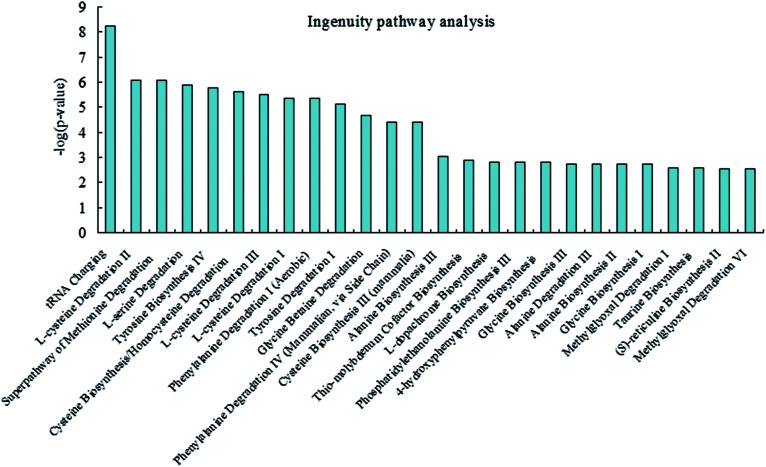
Ingenuity pathway analysis was performed on the significantly changed metabolites data.

To investigate and visualize the biological connectivity of the differentially expressed metabolites related to alkaline stress, the network-generating algorithm of IPA was used to maximize the interconnectivity of molecules based on all known connectivity in the online database. The result of the IPA biological function analysis (ESI Table S3†) is represented as a bar chart in Fig. 6. These molecular network representations clearly illustrate that the tRNA charging, l-cysteine degradation II, superpathway of methionine degradation, l-serine degradation, tyrosine biosynthesis IV, cysteine biosynthesis/homocysteine degradation, l-cysteine degradation III, l-cysteine degradation I, phenylalanine degradation I, and tyrosine degradation I appear to be the most significantly represented functional categories according to the differentially expressed metabolites dataset.

## Discussion

Fish are often exposed to environmental stress.^18,19^ The effects of excess alkalinity in CC fish are poorly understood. This information is urgently needed for further understanding the metabolic characteristics of alkaline stress tolerance. The high-throughput metabolomics can provide the tool for further investigation of the metabolic mechanism.^20–31^ Therefore, we examined the long-term effects of alkalinity stress on the CC. The metabolic profiles are studied by top-down thermometric analysis. Metabolic analyses conducted in the present study enhance the understanding on the impact of alkaline stress effects. The aim was to investigate the effects of exposure to a final treated alkalinity on the plasma metabolites of CC. Three groups of fish were susceptible to the alkaline concentration and the duration of exposure was 60 days. Plasma samples from alkalinity-exposed and control groups were profiled using our newly developed high-throughput UPLC-Q-TOF/MS combined with chemometrics in order to discover and enable the detection of differentially expressed metabolites. Our study revealed, for the first time, widespread disruption of differential metabolites in plasma of alkalinity-exposed fish, which could play a role in adaptation to the highly alkaline environment.

The results of this exploratory investigation show that the change in metabolism accurately reflects the alkaline conditions. Few metabolites and the variation in their levels with the alkalinity conditions were quantified; these metabolites displayed significant changes according to the UPLC-MS system results. Concurrently, 7 differential metabolites through UPLC/MS-based metabolomics were determined. In total, 23 related pathways could be potentially affected from alkalinity stress. Among the above pathways, 7 played important roles, namely, phenylalanine metabolism, glycine, serine and threonine metabolism, pyruvate metabolism, tyrosine metabolism, cysteine and methionine metabolism, aminoacyl-tRNA biosynthesis, and butanoate metabolism; these pathways may thus have key roles in regulating adversity development. Moreover, we highlighted the need for further understanding of the function of the numerous metabolites that were detected in CC plasma. The integrated pathway analysis was used to investigate and visualize biological connectivity of the differentially expressed metabolites related to alkalinity stress. The biological function analysis showed that the tRNA charging, l-cysteine degradation II, superpathway of methionine degradation, l-serine degradation, tyrosine biosynthesis IV, cysteine biosynthesis/homocysteine degradation, l-cysteine degradation III, l-cysteine degradation I, phenylalanine degradation I, and tyrosine degradation I appear to be the most significantly represented functional categories according to the differentially expressed metabolites dataset. These results shed new light on our understanding of the mechanisms responsible for ecological adaptation in CC fish.

Alkalinity exposure significantly affects metabolic profiles in the long-term alkalinity stress experiment. In our study, the use of high-throughput UPLC-Q-TOF/MS methods combined with chemometrics resulted in detection of differentially expressed metabolites in alkalinity-exposed fish that were associated with the phenylalanine metabolism, glycine, serine and threonine metabolism, pyruvate metabolism, tyrosine metabolism, cysteine and methionine metabolism, aminoacyl-tRNA biosynthesis, and butanoate metabolism pathways. In total, 7 metabolite structures were identified as significant markers of alkalinity exposure. This confirmed that our assays were appropriate for measuring physiological stress responses in CC. These metabolites are essential mediators regulating a diverse array of physiological systems and the disrupting of their metabolism warrants additional investigation on their effects. This study provides us useful information to explain the metabolic mechanism of alkaline stress tolerance.

## Conclusions

In summary, we utilized metabolic methods to identify the key signaling pathways and metabolic expression profiles associated with alkaline stress. We found 23 signaling pathways that could be potentially affected from alkalinity stress. Additionally, 7 key pathways related to the alkaline stress had been significantly enriched, indicating that these pathways are key for the adaptation to the highly alkaline environment. The generated information can be used to provide new insights into the alkalinity-mediated stress mechanisms of CC. In conclusion, our findings suggest that high-throughput UPLC-Q-TOF/MS methods combined with chemometrics are suitable for seeking the differentially expressed metabolites associated with environmental change and understanding the adaptation mechanism against extreme environmental stress. This study demonstrated that metabolomics could provide an insightful view of the small-molecule differential metabolites of CC under alkaline stress. It assists us in understanding adaptation under extreme environmental stress and will ultimately benefit future breeding programs for alkaline-tolerant fish.

## Conflicts of interest

The authors declare no competing financial interests.

## Supplementary Material

RA-008-C8RA01317A-s001
