# Supplementary material for: High-throughput metabolomics enables metabolite biomarkers and metabolic mechanism discovery of fish in response to alkalinity stress
Source: RSC Adv. 2018 Apr 19;8(27):14983–90. doi: 10.1039/c8ra01317a (PMC9079986; doi:10.1039/c8ra01317a)
Supplement: RA-008-C8RA01317A-s001 [file RA-008-C8RA01317A-s001.pdf]

Electronic Supplementary Material (ESI) for RSC Advances.
This journal is © The Royal Society of Chemistry 2018

Table S1. Biomarkers of CC after exposure to alkalinity stress identified by UPLC-MS metabolomics

No	VIP	Retention time (min)	m/z	Compound ID	Adducts	Formula	Mass Error (ppm)	Description	Anova (p)
1	14.7371	1.65	122.0269	HMDB00574	M+H	$C_3H_7NO_2S$	-1.69	L-Cysteine	0.0000
2	11.4794	7.48	182.0817	HMDB00158	M+H	$C_9H_{11}NO_3$	1.12	L-Tyrosine	0.0025
3	10.8457	2.77	165.0557	HMDB00205	M+H	$C_9H_8O_3$	0.81	Phenylpyruvic acid	0.0098
4	13.4087	3.72	101.0240	HMDB00060	M-H	$C_4H_6O_3$	-1.68	Acetoacetic acid	0.0093
5	11.9287	5.18	175.0239	HMDB00243	M-H	$C_3H_4O_3$	-2.10	Pyruvic acid	0.0001
6	11.7770	2.64	164.0859	HMDB00159	M-H	$C_9H_9NO_2$	-1.41	L-Phenylalanine	0.0017
7	9.45105	1.76	104.0499	HMDB00187	M-H	$C_3H_5NO_3$	0.33	L-Serine	0.0048

Table S2. Result from pathway analysis on alkalinity exposure group.

No.	Name	Total	Expected	Hits	Raw p	Impact
1	Phenylalanine, tyrosine and tryptophan biosynthesis	4	0.020911	3	3.50E-07	1
2	Phenylalanine metabolism	11	0.057506	3	1.42E-05	0.64815
3	Aminoacyl-tRNA biosynthesis	67	0.35026	4	0.000179	0.10256
4	Cysteine and methionine metabolism	29	0.15161	3	0.000302	0.1956
5	Glycine, serine and threonine metabolism	31	0.16206	3	0.00037	0.22606
6	Butanoate metabolism	22	0.11501	2	0.005151	0.10145
7	Ubiquinone and other terpenoid-quinone biosynthesis	3	0.015683	1	0.015613	0
8	Tyrosine metabolism	44	0.23002	2	0.019959	0.13743
9	Synthesis and degradation of ketone bodies	5	0.026139	1	0.025905	0.6
10	Cyanoamino acid metabolism	6	0.031367	1	0.031017	0
11	Taurine and hypotaurine metabolism	7	0.036594	1	0.036105	0
12	Thiamine metabolism	7	0.036594	1	0.036105	0
13	Sulfur metabolism	9	0.04705	1	0.046213	0.04167
14	Methane metabolism	9	0.04705	1	0.046213	0.4
15	Valine, leucine and isoleucine biosynthesis	13	0.067961	1	0.066158	0
16	Pantothenate and CoA biosynthesis	15	0.078417	1	0.075995	0
17	Citrate cycle (TCA cycle)	20	0.10456	1	0.1002	0.07184
18	Sphingolipid metabolism	21	0.10978	1	0.10498	0
19	Pyruvate metabolism	22	0.11501	1	0.10973	0.17409
20	Alanine, aspartate and glutamate metabolism	24	0.12547	1	0.11917	0
21	Glutathione metabolism	26	0.13592	1	0.12853	0.00371
22	Glycolysis or Gluconeogenesis	26	0.13592	1	0.12853	0.09956
23	Valine, leucine and isoleucine degradation	38	0.19866	1	0.1829	0

Table S3. Integrated pathway analysis of the differentially expressed metabolites related to alkalinity stress.

No.	Ingenuity Canonical Pathways	-log(p-value)	Ratio	Molecules
1	tRNA Charging	8.24	0.0488	L-phenylalanine,L-serine,L-tyrosine,L-cysteine
2	L-cysteine Degradation II	6.08	0.4	pyruvic acid,L-cysteine
3	Superpathway of Methionine Degradation	6.08	0.0455	pyruvic acid,L-serine,L-cysteine
4	L-serine Degradation	5.9	0.333	pyruvic acid,L-serine
5	Tyrosine Biosynthesis IV	5.76	0.286	L-phenylalanine,L-tyrosine
6	Cysteine Biosynthesis/Homocysteine Degradation	5.63	0.25	L-serine,L-cysteine
7	L-cysteine Degradation III	5.52	0.222	pyruvic acid,L-cysteine
8	L-cysteine Degradation I	5.34	0.182	pyruvic acid,L-cysteine
9	Phenylalanine Degradation I (Aerobic)	5.34	0.182	L-phenylalanine,L-tyrosine
10	Tyrosine Degradation I	5.12	0.143	acetoacetic acid,L-tyrosine
11	Glycine Betaine Degradation	4.68	0.087	pyruvic acid,L-serine
12	Phenylalanine Degradation IV (Mammalian, via Side Chain)	4.39	0.0625	L-phenylalanine,phenylpyruvic acid
13	Cysteine Biosynthesis III (mammalia)	4.39	0.0625	L-serine,L-cysteine
14	Alanine Biosynthesis III	3.03	0.333	L-cysteine
15	Thio-molybdenum Cofactor Biosynthesis	2.9	0.25	L-cysteine
16	L-dopachrome Biosynthesis	2.81	0.2	L-tyrosine
17	Phosphatidylethanolamine Biosynthesis III	2.81	0.2	L-serine
18	4-hydroxyphenylpyruvate Biosynthesis	2.81	0.2	L-tyrosine
19	Glycine Biosynthesis III	2.73	0.167	pyruvic acid
20	Alanine Degradation III	2.73	0.167	pyruvic acid
21	Alanine Biosynthesis II	2.73	0.167	pyruvic acid
22	Glycine Biosynthesis I	2.73	0.167	L-serine
23	Methylglyoxal Degradation I	2.6	0.125	pyruvic acid
24	Taurine Biosynthesis	2.6	0.125	L-cysteine
25	(S)-reticuline Biosynthesis II	2.55	0.111	L-tyrosine
26	Methylglyoxal Degradation VI	2.55	0.111	pyruvic acid
